# Copper-associated hepatitis in a patient with chronic myeloid leukemia following hematopoietic stem cell transplantation: A case report

**DOI:** 10.1097/MD.0000000000009041

**Published:** 2017-12-08

**Authors:** Ching-Fen Lee, Chi-Hua Chen, Yu-Chuan Wen, Tsung-Yen Chang, Ming-Wei Lai, Tang-Her Jaing

**Affiliations:** a Division of Clinical Pharmacy, Department of Pharmacy, Chang Gung Memorial Hospital, Taoyuan; b Department of Nursing, Chang Gung Memorial Hospital, Taoyuan; c Divisions of Hematology/Oncology, Department of Pediatrics, Chang Gung Children's Hospital, Chang Gung University, Taoyuan; d Division of Pediatric Gastroenterology, Department of Pediatrics, Chang Gung Children's Hospital, Chang Gung University, Taoyuan; e Liver Research Center, Department of Hepato-Gastroenterology, Chang Gung Memorial Hospital, Linkou, Taiwan.

**Keywords:** autoimmune hemolytic anemia, cholestatic liver disease, copper toxicity syndrome, drug-induced liver injury, hepatic graft-versus-host disease

## Abstract

**Rationale:**

We report a complicated case of cholestatic hepatitis with suspected autoimmune hemolytic anemia (AIHA) and copper toxicity syndrome after HSCT and donor lymphocyte infusion (DLI).

**Patient concerns:**

A 19-year-1-month-old girl presented with a history of CML. She underwent matched unrelated donor HSCT and donor lymphocyte infusion subsequently. Three months later, yellowish discoloration of the skin was found, which was accompanied by progressive itchy skin, easy fatigability, insomnia, and dark urine output. After admission, liver function disorders were observed.

**Intervention:**

Methylprednisolone was administered for suspected hepatic GVHD. Although abdominal sonography revealed no evidence of biliary tract obstruction and the viral hepatitis survey disclosed unremarkable findings; silymarin and ursodeoxycholic acid were administered to preserve the liver function. In addition, rituximab was prescribed for suspected AIHA. Because hyperbilirubinemia was progressive, mycophenolate and high-dose intravenous immunoglobulin were accordingly administered. As drug-induced liver injury cannot be excluded, all potential unconfirmed causes of drug-related hepatoxicity were discontinued.

**Diagnosis:**

In this case, the patient's history of shrimps and chocolate consumption led us to strongly suspect cholestatic hepatitis associated with copper toxicity syndrome. High 24-hour urine copper excretion and low serum zinc levels were also confirmed. Accordingly, d-penicillamine and zinc gluconate were administered.

**Outcomes:**

She succumbed to progressive hepatic failure and eventual multisystem organ failure 14 months after HSCT. No autopsy was performed.

**Lessons:**

This report described the combined effects of hepatic GVHD, AIHA, drugs, and copper toxicity on liver damage, and demonstrated the potential diagnostic challenges and treatment dilemmas associated with this disease.

## 1 Introduction

Cholestatic hepatitis is a rare complication of hematopoietic stem cell transplantation (HSCT). However, only a few reported cases have involved the successful treatment of cholestatic hepatitis after HSCT. Donor lymphocyte infusion (DLI) is used in post-HSCT relapses in many hematologic malignancies.^[1–4]^ The major complications of DLI are graft-versus-host disease (GVHD) and pancytopenia. Hepatic-variant GVHD should be considered in the differential diagnosis in DLI recipients with unexplained hepatitis.^[5]^

## 2 Case presentation

A 19-year-old girl presented with a history of chronic myeloid leukemia (CML) and cholestatic hepatitis that developed after DLI following HSCT. She had no history of hepatic dysfunction before donor lymphocyte infusion (DLI). She had developed a yellowish discoloration of skin on post-DLI day 95, and this was accompanied with progressive pruritus, high fatigability, insomnia, and dark urine output. She did not experience blurred vision, vomiting, diarrhea, abdominal mass, body weight loss, or skin rash. She was admitted for further evaluation under the suspicion of hepatic GVHD.

Although abdominal sonography revealed no evidence of biliary tract obstruction and a viral hepatitis serology survey yielded unremarkable findings, silymarin and ursodeoxycholic acid were administered to preserve liver function. After admission, laboratory values indicated marked cholestatic hepatitis, with a total serum bilirubin level of 9.6 mg/dL, direct bilirubin level of 5.4 mg/dL, aspartate aminotransferase (AST) level of 347 U/L, alanine aminotransferase (ALT) level of 559 U/L, and alkaline phosphatase (ALK-P) level of 247 U/L. Autoimmune hemolytic anemia (AIHA) was also suspected in this case, and hemogram data revealed a nucleated red blood cell count (RBC) of 2.0/100 white blood cells (WBC) and hemoglobin level of 75 g/L. Hence, intravenous methylprednisolone (2 mg/kg/d) was prescribed for suspected hepatic GVHD, and rituximab at 375 mg/m^2^ weekly for 4 weeks was administered for AIHA. Subsequently, mycophenolate and high-dose intravenous immunoglobulin (IVIG) were administered separately for progressive hyperbilirubinemia.

**Figure 1:**
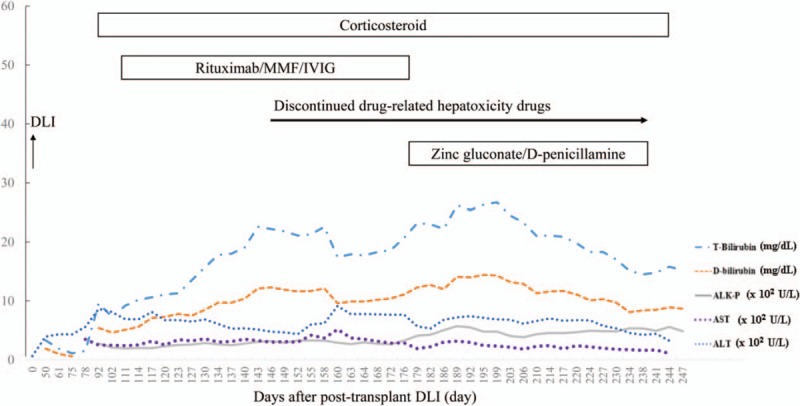
Changes of liver enzymes and bilirubin during treatment.

The patient's clinical course was summarized in Figure 1. The follow-up abdominal sonography disclosed homogenous liver echogenicity with no hepatomegaly or bile duct dilatation. The cytomegalovirus (CMV) DNA viral load was also evaluated to rule out CMV-related hepatitis. Although the laboratory data revealed progressive cholestatic hepatitis, the patient remained quite well except for the yellowish skin discoloration, and experienced improvement in her diffuse pruritus. A blood smear revealed polychromasia and erythroblastosis, indicating an ongoing hemolytic process. Because drug-induced liver injury could not be excluded, micafungin and all potential, unconfirmed causes of drug-related hepatoxicity were discontinued. However, the laboratory results continued to indicate an elevated nucleated RBC and abnormal liver function profiles. Therefore, oral prednisolone was replaced with intravenous methylprednisolone, and a vitamin K1 supplement was administered to address potential coagulopathy.

**Figure 2:**
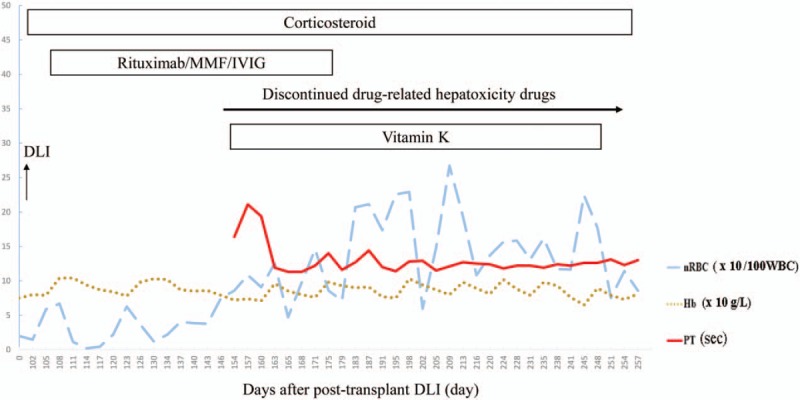
Changes of white blood cell count, hemoglobin, and platelets during treatment.

The clinical course of hemolysis anemia was summarized in Figure 2. The liver function disorder peaked on post-DLI day 164, when the AST and ALT levels increased to 516 and 913 U/L, respectively. Jaundice peaked on post- DLI day 199, when the total and direct bilirubin increased to levels as high as 26.2 and 14.1 mg/dL, respectively. Upon learning that the patient consumed large amounts of shrimps, other seafood and chocolate, we strongly suspected copper toxicity syndrome. Laboratory data showed a zinc level of 46.9 μg/dL (normal range: 70–120 μg/dL), ceruloplasmin level of 119.0 mg/dL (normal range: 20–60 mg/dL), and the 24-hour urine copper level of 204.6 μg/d (normal range: 15–50 μg/d). She was further diagnosed with cholestatic hepatitis associated copper toxicity syndrome, for which d-penicillamine and zinc gluconate were administered. However, d-penicillamine may facilitate the release of copper from the liver to the bloodstream, and could potentially cause a hemolytic crisis. Hence, d-penicillamine was tapered and eventually withdrawn. She succumbed to progressive hepatic failure and eventual multisystem organ failure 14 months after HSCT. No autopsy was performed.

## 3 Discussion

AIHA is a relatively uncommon complication and often treatment-refractory complication of HSCT.^[6]^ This condition can be idiopathic or secondary, and is classified as warm, cold, or mixed, according to the thermal range of the autoantibody.

AIHA may develop gradually or exhibit a fulminant onset with life-threatening anemia. Corticosteroids are considered first-line therapy for AIHA, and current opinions suggest that second-line therapy in primary warm AIHA should comprise splenectomy, rituximab, and any immunosuppressive drugs, in that sequence.^[7–9]^ Therefore, this case was treated with a combination of glucocorticoid and rituximab, followed by mycophenolate and high-dose IVIG, administered separately, for progressive hyperbilirubinemia.

Cholestatic liver disease (CLD) has a wide variety of etiologies. Drug-induced cholestasis is frequently included among the differential diagnoses in patients with cholestasis and normal hepatobiliary imaging. A pattern of cholestatic damage is characterized by an increase in ALP >3 times the upper limits of normal (ULN) and/or an ALT/ALP ratio <2. However, no specific or diagnostic clinical presentation, laboratory test, or histologic pattern is available to facilitate a diagnosis of CLD. Patients with prominent of features of hypersensitivity benefit from corticosteroid therapy.^[10]^ In the present case, all potential causes of drugs-related hepatoxicity were discontinued. However, the laboratory examination showed progressive hemolysis anemia.

In this case, the patient's history of shrimp and chocolate consumption led us to strongly suspect copper toxicity syndrome. The sudden onset of clinical signs in chronic copper toxicity is associated with the hemolytic crisis.^[11,12]^ Furthermore, her laboratory data revealed a low zinc level, high ceruloplasmin level, and excessive urinary copper excretion. Accordingly, we strongly suspected CLD associated with copper toxicity syndrome and administered d-penicillamine and zinc gluconate supplement to the patient.

## 4 Conclusion

This report therefore describes the combined liver-damaging effects of hepatic GVHD, AIHA, drug-related toxicity, and copper toxicity. This present case demonstrates the diagnostic challenge and therapeutic dilemmas associated with this condition. The fact that the severe liver complication might have been triggered or worsened by abnormal amounts of exogenous copper is speculative, although not surprising since severe cholestasis inhibits biliary copper excretion.
